# A Novel Technique for Detecting Antibiotic-Resistant Typhoid from Rapid Diagnostic Tests

**DOI:** 10.1128/JCM.00531-15

**Published:** 2015-04-16

**Authors:** Caoimhe Nic Fhogartaigh, David A. B. Dance, Viengmon Davong, Pisey Tann, Rattanaphone Phetsouvanh, Paul Turner, Sabine Dittrich, Paul N. Newton

**Affiliations:** a Lao—Oxford—Mahosot Hospital—Wellcome Trust Research Unit, Microbiology Laboratory, Vientiane, Lao PDR; b Public Health England, London, England, United Kingdom; c Centre for Tropical Medicine and Global Health, Nuffield Department of Medicine, Old Road Campus, University of Oxford, Oxford, England; d Cambodia—Oxford Medical Research Unit, Angkor Hospital for Children, Siem Reap, Cambodia

## Abstract

Fluoroquinolone-resistant typhoid is increasing. An antigen-detecting rapid diagnotic test (RDT) can rapidly diagnose typhoid from blood cultures. A simple, inexpensive molecular technique performed with DNA from positive RDTs accurately identified *gyrA* mutations consistent with phenotypic susceptibility testing results. Field diagnosis combined with centralized molecular resistance testing could improve typhoid management and surveillance in low-resource settings.

## TEXT

Antimicrobial resistance is a global problem of increasing concern. Over the past decade, strains of Salmonella enterica subsp. enterica serovar Typhi with ciprofloxacin MICs in the “intermediate” range have emerged and have been associated with adverse clinical outcomes and increased mortality (1, 2), leading to a lowering of ciprofloxacin breakpoints for *S*. Typhi (3, 4). Fluoroquinolone-resistant (FQR) *S*. Typhi is reported in 40 to 60% of typhoid cases (5, 6), with considerable geographical variation. Prevalence has reached 97% in southern Vietnam (5) and 90% in Cambodia (7), which is of great concern, as fluoroquinolones have become the mainstay of treatment for uncomplicated infection in areas where typhoid is endemic, except where the prevalence of resistance is known to be high, due to their ease of oral administration and low costs. Over 90% of FQR *S*. Typhi isolates are associated with point mutations in the *gyrA* gene, and the most common mutation is Ser83 → Phe (5).

Optimal detection of fluoroquinolone resistance by conventional techniques requires sophisticated laboratories that are able to use expensive consumables, have well-trained staff, and utilize quality assurance measures (8). With such capacities largely lacking in low- or middle-income countries, innovative approaches are needed. We demonstrated the utility of an antigen-detecting *S*. Typhi rapid diagnostic test (RDT) performed with blood culture fluid containing Gram-negative rods (GNRs) in typhoid diagnosis (9). As DNA extracts from RDTs and dried blood spots have been used for molecular surveillance of malaria and HIV drug resistance (10–12), we hypothesized that mutations in the *gyrA* gene could be detected from *S*. Typhi-positive RDTs, facilitating optimized treatment and public health interventions in remote areas. A pilot study was conducted to optimize methodologies, followed by a prospective, multicenter, hospital-based study that evaluated the sensitivity of *gyrA* mutation detection via RDTs and compared these results to those obtained with standard susceptibility testing.

### Pilot study.

**FIG 1:**
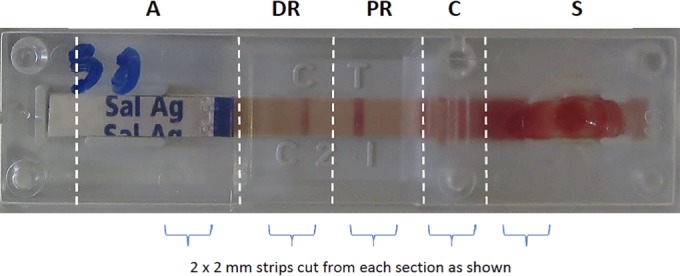
A Salmonella Typhi rapid diagnostic test. Each RDT strip is divided into five sections: sample (S), conjugate (C), proximal result (PR), distal result (DR), and absorption pads (A), which were cut into 2-mm strips to compare DNA extraction by elution or a column-based method.

Negative blood culture bottles (7 days) were seeded with ∼150 cells of *S*. Typhi NCTC 8385, reincubated, and inspected daily. A Gram stain was performed on turbid bottles to confirm the presence of GNRs. Ten bottles containing GNRs were used to inoculate 10 One-Step Salmonella Typhi antigen rapid detection kits (Standard Diagnostics, South Korea), which were used to optimize DNA extraction protocols (Fig. 1). Detection of the *gyrA* gene and its mutations was performed using previously described primers under slightly modified PCR conditions, followed by restriction fragment length polymorphism (RFLP) analysis (13). DNA from each section and extraction method underwent PCR as neat and diluted (1:10, 1:100, and 1:1,000) samples, plus 40 μg of bovine serum albumin (BSA; New England BioLabs) per reaction mixture to overcome inhibitors (14, 15). Subsequently, the intensities of bands on an agarose gel were compared, and the bands giving the greatest intensities were chosen as indicators for the optimal processing method. Wild-type *S*. Typhi NCTC 8385 and well-characterized strains with known *gyrA* mutations, provided by Oxford University Clinical Research Unit, Ho Chi Minh City, Vietnam (16), were used as controls.

The optimal protocol for DNA extraction was found to be the elution method (12), which consistently yielded more DNA than the column-based commercial kit (Qiagen, Germany). Sample or conjugate sections at the final dilution of 1:100 yielded similarly large amounts of DNA (Fig. 1).

### Prospective evaluations.

For the prospective evaluations, blood cultures taken with written and/or verbal informed consent from patients of all ages at Mahosot Hospital, Vientiane, Laos (May to October 2013) and children <15 years old at Angkor Hospital for Children (AHC), Siem Reap, Cambodia (June to October 2013) were included. Ethical clearance was granted by the Oxford Tropical Research Ethics Committee, University of Oxford, United Kingdom, and local ethics committees.

Positive blood culture fluid containing GNRs was used to perform the *S*. Typhi RDT, and positive RDTs were individually stored in ziplock bags at 4°C until extraction and *gyrA* PCR-RFLP. The positive RDT samples from Cambodia were transported to the Mahosot laboratory at ambient temperature (maximum 36 h of travel) (17). Antimicrobial susceptibility testing of confirmed isolates was performed according to published guidelines, including disk-diffusion tests (Oxoid, United Kingdom) for ciprofloxacin (5 μg) and nalidixic acid (30 μg) and MIC testing via Etest (bioMérieux, France) for ciprofloxacin (18, 19). RDTs were performed on GNR-containing blood cultures from 172 patients (Laos, *n* = 136; Cambodia, *n* = 36). RDTs were positive for 38 patients (Laos, 28/136 [20.6%]; Cambodia, 10/36 [27.8%]), including 31 *S*. Typhi and 7 non-*S*. Typhi group D salmonellae. Available RDT samples (from total RDTs, 31/38 [81.6%]; *S*. Typhi, 25/31 [80.6%]; group D salmonellae, 6/7 [85.7%]) were tested under the optimized *gyrA* detection protocol. The median time from RDT to extraction was 42 days (range, 8 to 134 days). All *S*. Typhi *gyrA* results showed 100% agreement with phenotypic susceptibilities, including 7 FQR cases: 1/19 (5.3%) from Laos had a single mutation at codon 83; 4/6 (66.7%) from Cambodia had a single mutation at codon 83, and 2/6 (33.3%) had double mutations at codons 83 and 87 (Table 1).

**TABLE 1:** Results of PCR and RFLP for *gyrA* mutations in *S*. Typhi and corresponding ciprofloxacin MIC results^*a*^

Lab. ID no.	Country	CIP MIC (μg/ml)	Interpretation (S/I/R)	PCR-RFLP result (WT or mutated codon[s])
2956	Laos	0.012	S	WT
2957	Laos	0.008	S	WT
2965	Laos	0.016	S	WT
3009	Laos	0.016	S	WT
27416	Laos	0.023	S	WT
3624	Laos	0.25	I	83
3770	Laos	0.016	S	WT
2986	Laos	0.016	S	WT
27545	Laos	0.016	S	WT
3823	Laos	0.023	S	WT
27986	Laos	0.012	S	WT
27987	Laos	0.012	S	WT
28247	Laos	0.012	S	WT
28257	Laos	0.012	S	WT
28317	Laos	0.016	S	WT
28373	Laos	0.008	S	WT
28403	Laos	0.032	S	WT
28412	Laos	0.008	S	WT
3862	Laos	0.023	S	WT
3286	Cambodia	0.5	I	83, 87
3472	Cambodia	0.5	I	83
3473	Cambodia	0.5	I	83
3489	Cambodia	0.5	I	83, 87
3543	Cambodia	0.5	I	83
4402	Cambodia	0.25	I	83

a Isolates were identified by using the API 20E test (Laos) or an in-house biochemical test set (Cambodia) and Salmonella Omni-O, O9, Vi, and Hd antisera (Pro-lab Diagnostics, United Kingdom) at both sites. Every PCR-RFLP investigation included the following controls to guide interpretation. S83F (*gyrA* codon 83): nalidixic acid (NA) MIC of 256 μg/ml, ofloxacin (OFX) MIC of 0.38 μg/ml, ciprofloxacin (CIP) MIC of 0.125 μg/ml. D87A (*gyrA* codon 87): NA MIC of 48 μg/ml, OFX MIC of 0.19 μg/ml, CIP MIC of 0.094 μg/ml. S80I (*gyrA* codons 83 and 87; *parC* codon 80): NA MIC of 256 μg/ml, OFX MIC of 16 μg/ml, CIP MIC of 8 μg/ml. S, sensitive; I, intermediate; R, resistant; WT, wild type.

The method, albeit with a small sample size, was therefore 100% sensitive and 100% specific in detecting *gyrA* mutations from RDT-derived DNA to predict FQR *S*. Typhi.

Limitations of the study are that the RDT also detects other group D salmonellae that share the O9 antigen, and the current PCR-RFLP only detects *gyrA* mutations. An additional molecular test to confirm *S*. Typhi (20) could be incorporated into a multiplex or nested PCR, which could be further developed to include primers for other resistance genes, should these become more prevalent, with the option of subsequent sequencing to provide more-detailed molecular epidemiology data on resistance and phylogeny.

After RDTs are conducted on *S*. Typhi isolates in the field, the small RDT package can be conveniently transported to a central reference laboratory, eliminating the risk of injuries from sharp medical instruments (needles) and blood-borne infections from transport of blood culture bottles. Although RDTs contain fewer viable organisms than bacterial colonies (21), inactivation techniques, used successfully for other bacterial pathogens (22), could further decrease risks and make our approach more applicable to field conditions. As standard susceptibility testing of blood culture isolates takes two additional days, if RDTs are shipped speedily, the PCR-RFLP can provide accelerated results to guide patient management within the same day. This could be particularly useful during outbreak investigations. However, as rapid transport depends on the local infrastructure, larger feasibility studies are needed to investigate the real-life impact of molecular resistance testing on patient or outbreak management. Even if patient management cannot be directly influenced due to transport constraints, batched results will provide valuable data for FQR surveillance to inform public health guidelines and treatment policies.

In conclusion, with the increasing global frequency of drug resistance, use of molecular markers from RDTs represents an innovative, accurate, and potentially cost-effective method for both individual patient diagnosis and public health surveillance in countries without accessible clinical microbiology laboratories.

## References

[1] Crump JA, Kretsinger K, Gay K, Hoekstra RM, Vugia DJ, Hurd S, Segler SD, Megginson M, Luedeman LJ, Shiferaw B, Hanna SS, Joyce KW, Mintz ED, Angulo FJ, Emerging Infections Program FoodNet, NARMS Working Groups. 2008 Clinical response and outcome of infection with Salmonella enterica serotype Typhi with decreased susceptibility to fluoroquinolones: a United States FoodNet multicenter retrospective cohort study. Antimicrob Agents Chemother 52:1278–1284. doi:10.1128/AAC.01509-07. 18212096 PMC2292528

[2] Parry CM, Vinh H, Chinh NT, Wain J, Campbell JI, Hien TT, Farrar JJ, Baker S 2011 The influence of reduced susceptibility to fluoroquinolones in Salmonella enterica serovar Typhi on the clinical response to ofloxacin therapy. PLoS Negl Trop Dis 5:e1163. doi:10.1371/journal.pntd.0001163. 21713025 PMC3119645

[3] Clinical and Laboratory Standards Institute. 2013 Performance standards for antimicrobial disk susceptibility tests; 23rd information supplement, CLSI document M100-S23 Clinical and Laboratory Standards Institute, Wayne, PA.

[4] Humphries RM, Fang FC, Aarestrup FM, Hindler JA 2012 In vitro susceptibility testing of fluoroquinolone activity against Salmonella: recent changes to CLSI standards. Clin Infect Dis 55:1107–1113. doi:10.1093/cid/cis600. 22752519

[5] Chau TT, Campbell JI, Galindo CM, Van Minh Hoang N, Diep TS, Nga TT, Van Vinh Chau N, Tuan PQ, Page AL, Ochiai RL, Schultsz C, Wain J, Bhutta ZA, Parry CM, Bhattacharya SK, Dutta S, Agtini M, Dong B, Honghui Y, Anh DD, Canh do G, Naheed A, Albert MJ, Phetsouvanh R, Newton PN, Basnyat B, Arjyal A, La TT, Rang NN, Phuong le T, Van Be Bay P, von Seidlein L, Dougan G, Clemens JD, Vinh H, Hien TT, Chinh NT, Acosta CJ, Farrar J, Dolecek C 2007 Antimicrobial drug resistance of Salmonella enterica serovar Typhi in Asia and molecular mechanism of reduced susceptibility to the fluoroquinolones. Antimicrob Agents Chemother 51:4315–4323. doi:10.1128/AAC.00294-07. 17908946 PMC2167998

[6] Ochiai RL, Acosta CJ, Danovaro-Holliday MC, Baiqing D, Bhattacharya SK, Agtini MD, Bhutta ZA, Canh do G, Ali M, Shin S, Wain J, Page AL, Albert MJ, Farrar J, Abu-Elyazeed R, Pang T, Galindo CM, von Seidlein L, Clemens JD, Domi Typhoid Study Group. 2008 A study of typhoid fever in five Asian countries: disease burden and implications for controls. Bull World Health Organ 86:260–268. doi:10.2471/BLT.06.039818. 18438514 PMC2647431

[7] Emary K, Moore CE, Chanpheaktra N, An KP, Chheng K, Sona S, Duy PT, Nga TV, Wuthiekanun V, Amornchai P, Kumar V, Wijedoru L, Stoesser NE, Carter MJ, Baker S, Day NP, Parry CM 2012 Enteric fever in Cambodian children is dominated by multidrug-resistant H58 Salmonella enterica serovar Typhi with intermediate susceptibility to ciprofloxacin. Trans R Soc Trop Med Hyg 106:718–724. doi:10.1016/j.trstmh.2012.08.007. 23122884

[8] Peacock SJ, Newton PN 2008 Public health impact of establishing the cause of bacterial infections in rural Asia. Trans R Soc Trop Med Hyg 102:5–6. doi:10.1016/j.trstmh.2007.06.004. 17619030

[9] Castonguay-Vanier J, Davong V, Bouthasavong L, Sengdetka D, Simmalavong M, Seupsavith A, Dance DA, Baker S, Le Thi Phuong T, Vongsouvath M, Newton PN 2013 Evaluation of a simple blood culture amplification and antigen detection method for diagnosis of Salmonella enterica serovar Typhi bacteremia. J Clin Microbiol 51:142–148. doi:10.1128/JCM.02360-12. 23100346 PMC3536227

[10] Bertagnolio S, Parkin NT, Jordan M, Brooks J, Garcia-Lerma JG 2010 Dried blood spots for HIV-1 drug resistance and viral load testing: a review of current knowledge and WHO efforts for global HIV drug resistance surveillance. AIDS Rev 12:195–208. http://www.aidsreviews.com/files/2010_12_4_195-208.pdf. 21179184

[11] Cnops L, Boderie M, Gillet P, Van Esbroeck M, Jacobs J 2011 Rapid diagnostic tests as a source of DNA for Plasmodium species-specific real-time PCR. Malar J 10:67. doi:10.1186/1475-2875-10-67. 21435256 PMC3075219

[12] Morris U, Aydin-Schmidt B, Shakely D, Martensson A, Jornhagen L, Ali AS, Msellem MI, Petzold M, Gil JP, Ferreira PE, Bjorkman A 2013 Rapid diagnostic tests for molecular surveillance of Plasmodium falciparum malaria: assessment of DNA extraction methods and field applicability. Malar J 12:106. doi:10.1186/1475-2875-12-106. 23510231 PMC3605315

[13] Giraud E, Brisabois A, Martel JL, Chaslus-Dancla E 1999 Comparative studies of mutations in animal isolates and experimental in vitro- and in vivo-selected mutants of Salmonella spp. suggest a counterselection of highly fluoroquinolone-resistant strains in the field. Antimicrob Agents Chemother 43:2131–2137. 10471553 10.1128/aac.43.9.2131 PMC89435

[14] Al-Soud WA, Radstrom P 2001 Purification and characterization of PCR-inhibitory components in blood cells. J Clin Microbiol 39:485–493. doi:10.1128/JCM.39.2.485-493.2001. 11158094 PMC87763

[15] Fredricks DN, Relman DA 1998 Improved amplification of microbial DNA from blood cultures by removal of the PCR inhibitor sodium polyanetholesulfonate. J Clin Microbiol 36:2810–2816. 9738025 10.1128/jcm.36.10.2810-2816.1998 PMC105069

[16] Baker S, Duy PT, Nga TV, Dung TT, Phat VV, Chau TT, Turner AK, Farrar J, Boni MF 2013 Fitness benefits in fluoroquinolone-resistant Salmonella Typhi in the absence of antimicrobial pressure. eLife 2:e01229. doi:10.7554/eLife.01229. 24327559 PMC3857714

[17] Blacksell SD, Khounsy S, Phetsouvanh R, Newton PN 2006 A simple and inexpensive container for the transport of biological specimens in limited resource situations. Trans R Soc Trop Med Hyg 100:1084–1086. doi:10.1016/j.trstmh.2006.03.005. 16808939 PMC7610911

[18] Clinical and Laboratory Standards Institute. 2012 Methods for dilution antimicrobial susceptibility tests for bacteria that grow aerobically; approved standard, 9th ed CLSI document M07-A9 Clinical and Laboratory Standards Institute, Wayne, PA.

[19] Clinical and Laboratory Standards Institute. 2013 Performance standards for antimicrobial susceptibility testing; 23rd informational supplement. CLSI document M100-S23 Clinical and Laboratory Standards Institute, Wayne, PA.

[20] Zhou L, Pollard AJ 2010 A fast and highly sensitive blood culture PCR method for clinical detection of Salmonella enterica serovar Typhi. Ann Clin Microbiol Antimicrob 9:14. doi:10.1186/1476-0711-9-14. 20403166 PMC2873252

[21] Smit PW, Elliott I, Peeling RW, Mabey D, Newton PN 2014 An overview of the clinical use of filter paper in the diagnosis of tropical diseases. Am J Trop Med Hyg 90:195–210. doi:10.4269/ajtmh.13-0463. 24366501 PMC3919219

[22] Burger M, Raskin S, Brockelt SR, Amthor B, Geiss HK, Haas WH 1998 DNA fingerprinting of Mycobacterium tuberculosis complex culture isolates collected in Brazil and spotted onto filter paper. J Clin Microbiol 36:573–576. 9466780 10.1128/jcm.36.2.573-576.1998 PMC104581

